# The effect of imipramine, ketamine, and zinc in the mouse model of depression

**DOI:** 10.1007/s11011-015-9709-6

**Published:** 2015-07-10

**Authors:** Andrzej Wróbel, Anna Serefko, Piotr Wlaź, Ewa Poleszak

**Affiliations:** Second Department of Gynecology, Medical University of Lublin, Lublin, Poland; Department of Applied Pharmacy, Medical University of Lublin, Chodźki 1, PL 20-093 Lublin, Poland; Department of Animal Physiology, Institute of Biology and Biochemistry, Maria Curie-Skłodowska University, Lublin, Poland

**Keywords:** Dexamethasone, Antidepressant-like activity, Forced swim test, Mice

## Abstract

Relationship between the chronic and excessive exposure to glucocorticoids and the development of psychiatric disorders, including depression, has been described in the literature. We decided to investigate whether a combination of agents with antidepressant activity (i.e., imipramine, ketamine, and Zn^2+^) may influence/reverse the depressogenic effect of dexamethasone therapy. The antidepressant-like effect was assessed by the forced swim test in adult mice. The inhibitory activity of dexamethasone was dose-dependent: only the highest tested dose of the glucocorticoid (i.e., 64 μg/kg) given as a single injection increased immobility time, whereas 16 μg/kg/day of dexamethasone administered repeatedly (for 14 days) induced a significant alteration in animal behavior. Both the acute or sub-chronic administration of the active doses of imipramine (10 mg/kg), Zn^2+^ (30 mg/kg), and ketamine (30 mg/kg), and the combinations of their per se inactive doses reversed the inhibitory activity of dexamethasone (16 μg/kg/day) administered for 14 consecutive days. Whereas a single injection of an inhibitory dose of dexamethasone (64 μg/kg) was not able to abolish the antidepressant effect of imipramine (5 mg/kg), Zn^2+^ (10 mg/kg), and imipramine-Zn^2+^ combination (2.5 and 5 mg/kg, respectively) given once a day for 14 consecutive days. Our findings indicate that the chronic dexamethasone injection procedure has some potential as an animal model of depression and they further support the theory of interplay between glutamatergic neurotransmission and the chronic or excessive exposition to glucocorticoids.

## Introduction

Organism reaction to stress is an important adaptive response that mobilises the organism and re-establishes homeostasis. Neuronal, endocrine and immune mechanisms are turned on, including activation of the hypothalamic–pituitary–adrenal (HPA) axis, and an increased secretion of glucocorticoids is noted. Though indispensable for coping with stressful events, glucocorticoids may have a negative effect on both structure and function of brain cells. The hippocampus, as highly abundant in glucocorticoid receptors, seems to be particularly vulnerable to stress (Bosch et al. 2012; McEwen et al. 1969). In consequence, chronic and excessive exposure to glucocorticoids may lead to development of psychiatric disorders, including depression. In fact, depression was reported in psychiatrically healthy patients receiving chronic high doses of glucocorticoids as well as in the people with Cushing’s syndrome (Brown et al. 1999). On the other hand, patients with endogenous depression present HPA axis hyperactivity (elevated cortisol and adrenocorticotropin hormone levels, enlargement of adrenal glands) and failure to suppress cortisol in response to the dexamethasone suppression test (Stetler and Miller 2011). Some of the antidepressant agents (i.e., desipramine, fluoxetine) influenced the neuroendocrine substrates that regulate cortisol secretion (Antonioli et al. 2012; Okuyama-Tamura et al. 2003). Though it is still not obvious whether the high cortisol level seen in depressed patients plays a causal role in depression or whether it is a “by-product” of depression. After exposure to chronic stress or administration of glucocorticoids, animals show behavioral changes associated with depression such as: anhedonia, food intake disorder, cognitive deficits, decrease in sexual activity, increased immobility time in the forced swim test (FST) and the tail suspension test (TST), elevated anxiety levels (Feldmann et al. 2008; Gorzalka and Hanson 1998; Gourley et al. 2008; Gregus et al. 2005; Zhao et al. 2008). Similarly, biochemical (altered glucocorticoid receptor concentration, increased glutamate level, serotonin deficiency, modulation of 5-HT1A and 5-HT2A receptors function) (Ago et al. 2008; Fernandes et al. 1997; Li et al. 2014), morphological (neuronal remodeling in the hippocampus, amygdala and medial prefrontal cortex) (Vyas et al. 2002; Wellman 2001; Woolley et al. 1990), and functional changes (reduction of neurogenesis in the dentate gyrus of the hippocampus) (Pham et al. 2003) are observed. It has been shown that the glucocorticoid receptor antagonists may ameliorate the behavioral and neurochemical alterations of depression (Iijima et al. 2010). Moreover, Kitayama et al. (1988) have found that that chronic treatment with imipramine may increase the regulation of glucocorticoid receptor. Based on the research of Biagini et al. (1993), imipramine also antagonized the behavioral and endocrine responses induced by repeated exposure to unpredictable stress in the rat model. It can, therefore, be assumed that the modelled depression and the associated increase in corticosterone level may be controlled by this tricyclic antidepressant.

Chronic corticosterone-injected rodents have been proposed as an animal model of depression that mimics the dysfunction of the HPA axis in depression. Several studies have indicated that the repeated corticosterone administration elicit an increase in immobility behavior during the FST and TST in male and female rodents without affecting their overall locomotion (Ago et al. 2008; Gregus et al. 2005; Johnson et al. 2006; Koike et al. 2013). Though, Meyer et al. (2012) pointed out that the chronic corticosterone-injected model is not suitable for the adolescent rats.

The results of our previous experiments revealed that the single and 7-day administration of dexamethasone lead to a depressogenic effect in adult mice (Wróbel et al. 2014). Similarly, Li et al. (2014) reported that neonatal dexamethasone exposure caused depression-like behavior in juvenile mice, suggesting that this paradigm may be a new animal model of pediatric depression. Though there are many studies on the effect of antidepressants in naïve animals, it seems to be more appropriate to evaluate the effects of the drugs in models that mimic symptoms of human depression. Therefore, we decided to investigate whether a combination of agents with antidepressant activity (i.e., imipramine—a classic tricyclic antidepressant, ketamine—a noncompetitive NMDA receptor antagonist, and Zn^2+^—the divalent inorganic inhibitor of the distinct binding site at the NMDA receptor complex) may influence/reverse the depressogenic effect of dexamethasone therapy.

## Materials and methods

### Animals

The study was conducted on adult male Albino Swiss mice (weighting initially 18–25 g). A natural light/dark cycle, temperature of 23 ± 1 °C and humidity of 50–60 % were maintained. Food and water were provided ad libitum. All experimental procedures were carried out between 8 a.m. and 1 p.m. Mice were experimentally naïve and tested once. Ninety four groups of animals were used in the study, each one consisted of 12–15 animals. All procedures were conducted in accordance with the European Communities Council Directive of 22 September 2010 (2010/63/EU) and Polish legislation acts concerning animal experimentations. The experimental procedures and protocols were approved by the First Local Ethics Committee at the Medical University of Lublin.

### Drugs

The following drugs were used: dexamethasone (Jelfa, Poland), imipramine (Polpharma, Poland), zinc hydroaspartate (Farmapol, Poznań, Poland), and ketamine (Parke-Davis, Berlin, Germany). Imipramine, zinc hydroaspartate, and ketamine were administered intraperitoneally (i.p.), whereas dexamethasone was given subcutaneously (s.c.), at 10 ml/kg in water.

### Locomotor activity

The locomotor activity of animals was assessed with the aid of a Digiscan apparatus: an Optical Animal Activity Monitoring System (Omnitech Electronics, Inc., Columbus, Ohio, USA). Activity chambers consisting of clear acrylic open field boxes were located in a room lit by a dim red light. The Digiscan system monitored animal locomotor activity via a grid of invisible infrared light beams. Cumulative counts of beams interruptions were recorded in 15 min intervals using OMNIPRO software. Prior to behavioral analysis, subjects were placed into activity chambers for a 15-min habituation period. Experiments were performed in a sound-proof room. Horizontal activity was assessed. This was defined as the total number of beam interruptions that occurred in the horizontal sensor during one hour of measurement.

### Forced swim test

The studies were carried out on mice according to the method of Porsolt et al. (1977). Mice were placed individually into glass cylinders (height 25 cm, diameter 10 cm) containing 10 cm of water, maintained at 23–25 °C. Animals were removed and returned to their home cages after 1 min in water. Twenty four hours later, they were again placed in the cylinder. Mice were left there for 6 min. After the first 2 min, the total duration of immobility was measured during the following 4-min test. The mouse was judged to be immobile when it remained floating passively, performing slow motion movements to keep its head above the water.

### Experiments

#### Experiment 1: Determination of the effect of dexamethasone on the immobility time of mice in the FST and their spontaneous locomotor activity

Dexamethasone was given in a single dose (4, 16 or 64 μg/kg) or for 14 days at the doses of 4 or 16 μg/kg/day. Locomotor activity or immobility time was registered 3.5 h after a single or 48 h after the last injection (in the 14-day experiment) of dexamethasone. The time intervals between administration of dexamethasone and the behavioral test were selected on the basis of our previous studies on the behavioral effects of dexamethasone (Wróbel et al. 2014) and were confirmed in the preliminary experiments.

#### Experiment 2: Evaluation of the influence of a sub-chronic administration of zinc or the acute injections of imipramine or ketamine, and their combinations on the 14-day dexamethasone treatment in the FST

Dexamethasone was given at the dose of 16 μg/kg/day for 14 days. Locomotor activity or immobility time were registered 48 h after the last injection of the glucocorticoid. Antidepressant agents were given in the following scheme: imipramine (5 or 10 mg/kg) and ketamine (30 mg/kg) were administered only once 1 h and 30 min prior to the behavioral tests, respectively, and zinc hydroaspartate (15 or 30 mg/kg) was injected three times (sub-chronic treatment, i.e., 24, 5 and 1 h before the behavioral tests). Dosage of zinc referred to pure zinc ions. The doses of all substances as well as the time interval between their administration and the behavioral tests were taken from the literature (Browne and Lucki 2013; Nowak et al. 2003; Wróbel et al. 2014) and were confirmed/adjusted in our laboratory in preliminarily experiments.

#### Experiment 3: Evaluation of the influence of a chronic administration of imipramine, zinc, and their combination on a single injection of dexamethasone in the FST

Imipramine (2.5 or 5 mg/kg/day) and zinc hydroaspartate (5 or 10 mg/kg/day) were given for 14 days, while dexamethasone (64 μg/kg) was administered only once. Locomotor activity or immobility time were registered 48 h after the last injection of imipramine and/or zinc hydroaspartate. Dexamethasone was given 3.5 h prior to the behavioral tests.

#### Experiment 4: Evaluation of the influence of a chronic administration of imipramine and zinc given in combination with ketamine on a single injection of dexamethasone in the FST

Per se inactive doses of imipramine (2.5 mg/kg/day) and zinc hydroaspartate (5 mg/kg/day), were given for 14 days. An inhibitory dose of dexamethasone (64 μg/kg) and an antidepressant dose of ketamine (30 mg/kg) were administered only once. Locomotor activity or immobility time were registered 48 h after the last injection of imipramine and/or zinc hydroaspartate. Dexamethasone and ketamine were given 3.5 h and 14 days prior to the behavioral tests, respectively.

### Statistical methods

The obtained data were assessed by the *t*-test, one-way or two-way analysis of variance (ANOVA) followed by Bonferroni’s post hoc test, depending on the experimental design. All results are presented as the means ± standard error of the mean (SEM). *P* < 0.05 was considered a statistically significant difference.

## Results

### The effect of dexamethasone on the immobility time of mice in the FST and their spontaneous locomotor activity

**Fig. 1:**
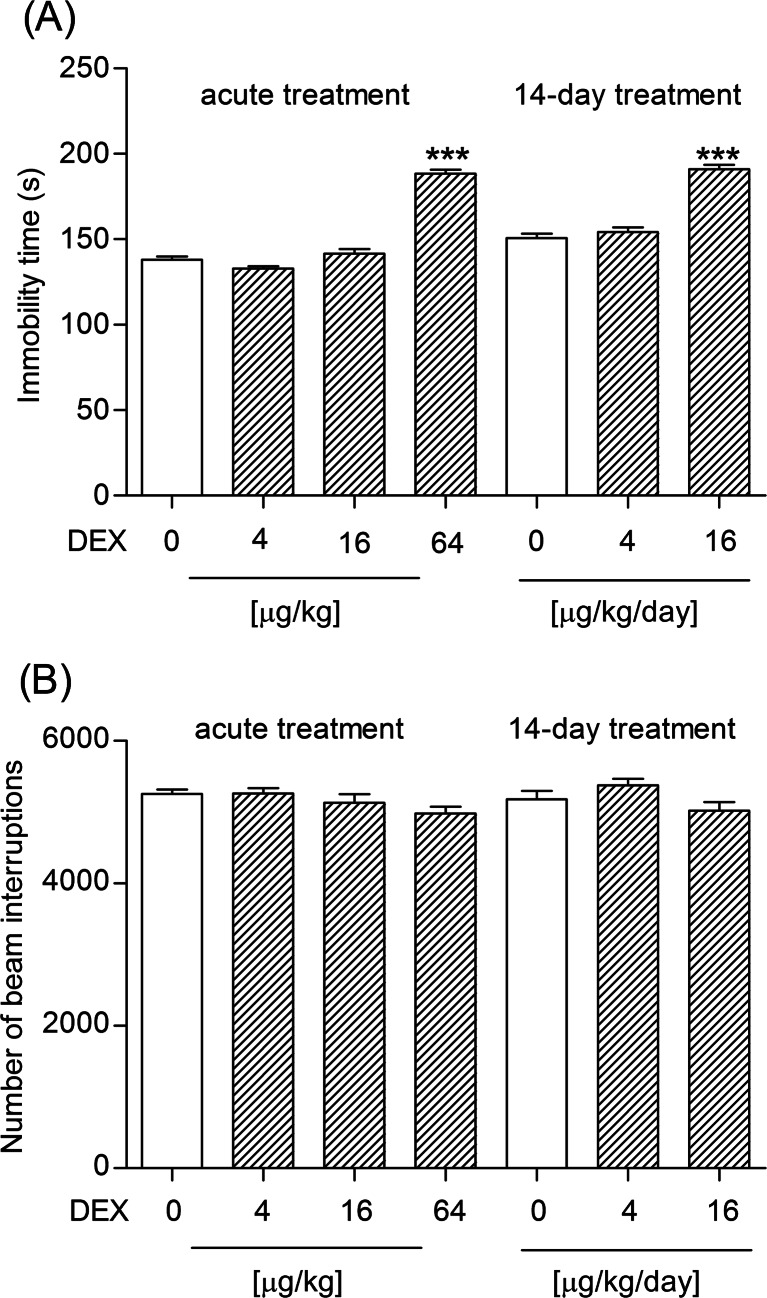
Effect of acute (4, 16 or 64 μg/kg) and 14-day (4 or 16 μg/kg) treatment with dexamethasone on **a** the behavior of mice in the FST and **b** the spontaneous locomotor activity in mice. Dexamethasone was given s.c. The behavioral tests were performed 3.5 or 48 h after a single or the last (in the chronic experiment) injection of dexamethasone, respectively. The values represent the mean + SEM (*n* = 15 mice per group). ***P < 0.001 versus control (s.c.) (Bonferroni’s post hoc test)

Sixty four μg/kg of dexamethasone given as a single injection considerably increased the immobility time of the animals in the FST (one-way ANOVA: *F*_3,56_ = 141.7, *P* < 0.0001). The doses of 4 and 16 μg/kg appeared to be too low to influence the behavior of mice in this experiment. Animals subjected to a 14-day treatment with dexamethasone at a dose of 16 μg/kg/day spent significantly less time swimming in comparison to the control group (one-way ANOVA: *F*_2,42_ = 71.39, *P* < 0.0001; Fig. 1a). Neither single nor repeated (14-day) administration of dexamethasone in the tested doses significantly changed the spontaneous locomotor activity of mice (one-way ANOVA: *F*_3,56_ = 2.100, *P* = 0.1105 and *F*_2,42_ = 2.664, *P* = 0.0815 for the single and repeated administration, respectively; Fig. 1b).

### The influence of a sub-chronic administration of zinc or the acute injections of imipramine or ketamine, and their combinations on the 14-day dexamethasone treatment in the FST

**Fig. 2:**
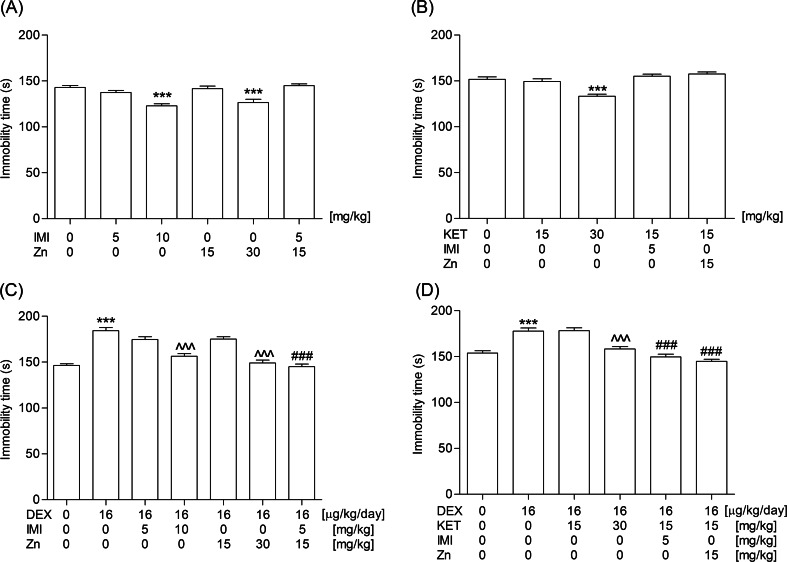
Influence of the acute injections of imipramine (5 or 10 mg/kg; IMI), zinc (15 or 30 mg/kg; Zn), ketamine (15 or 30 mg/kg; KET), and their combinations on the 14-day dexamethasone (16 μg/kg/day; DEX) treatment in the FST in mice. The behavioral test was performed 48 h after the last s.c. injection of dexamethasone. Imipramine and ketamine were administered i.p. only once 1 h and 30 min prior to the behavioral tests, respectively, and zinc hydroaspartate was injected i.p. three times (i.e., 24, 5 and 1 h before the behavioral tests). The experiments were performed on the naïve (**a** and **b**) and dexamethasone-treated (**c** and **d**) and mice. The values represent the mean + SEM (*n* = 13–15 mice per group). ^^^*P* < 0.001 versus dexamethasone (Bonferroni’s post hoc test), ^###^ *P* < 0.001 versus dexamethasone and respective antidepressant(s) (Bonferroni’s post hoc test), ***P < 0.001 versus control (*t*-test or Bonferroni’s post hoc test)

As illustrated in Fig. 2c and d, the active doses of the tested antidepressant agents (i.e., 10, 30, and 30 mg/kg for imipramine, Zn^2+^, and ketamine, respectively), that were selected on the naïve animals, abolished an inhibitory effect of the repeated treatment with dexamethasone. The combinations of imipramine (5 mg/kg) with zinc (15 mg/kg), imipramine (5 mg/kg) with ketamine (15 mg/kg), and zinc (15 mg/kg) with ketamine (15 mg/kg) reversed the inhibitory activity of dexamethasone administered at a dose of 16 μg/kg once a day for 14 consecutive days (two-way ANOVA: *F*_1,54_ = 11.22, *P* = 0.0015 for imipramine-zinc interaction; one-way ANOVA: *F*_2,42_ = 24.60, *P* < 0.0001 and *F*_2,42_ = 39.06, *P* < 0.0001 for imipramine-ketamine and zinc-ketamine combinations, respectively). These combinations appeared to be not active in the naïve mice (Fig. 2a and b).

### Influence of a chronic administration of imipramine, zinc, and their combination on a single injection of dexamethasone in the FST

**Fig. 3:**
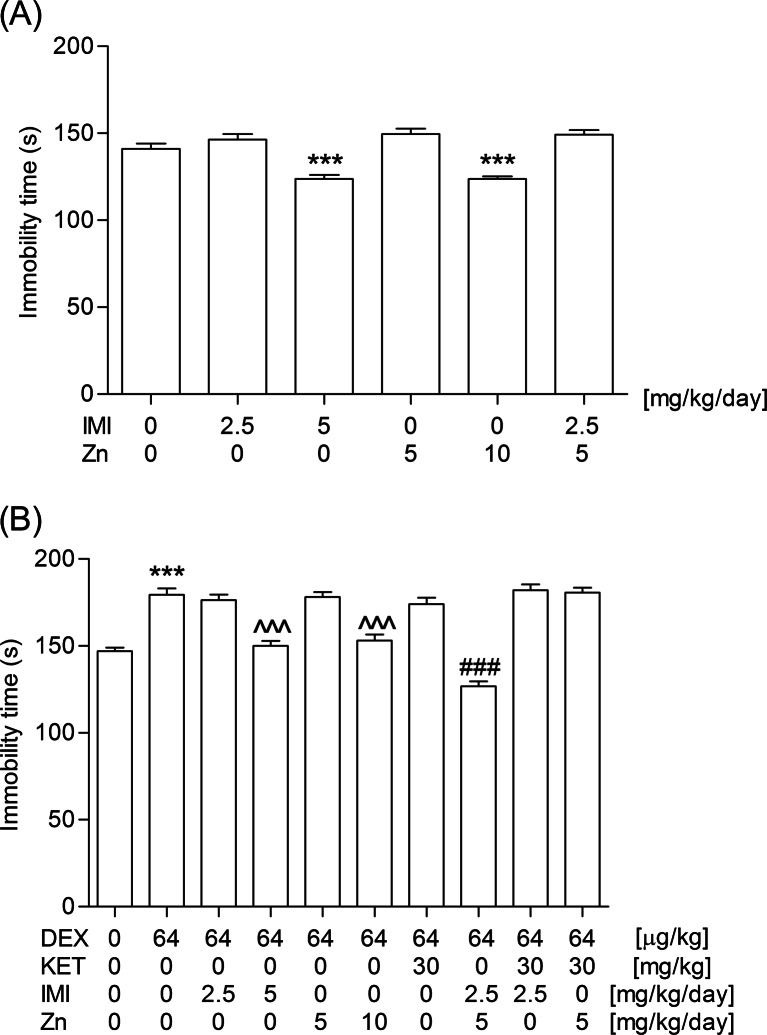
Influence of a single injection of dexamethasone (64 μg/kg; DEX) on the 14-day administration of imipramine (2.5 or 5 mg/kg/day; IMI), zinc (5 or 10 mg/kg/day; Zn), their combination, and the combination with a single injection of ketamine (30 mg/kg; KET) in the FST. Dexamethasone was given s.c. 3.5 h before the behavioral test. The behavioral test was performed 48 h after the last i.p. injection of imipramine and/or zinc and 14 days after i.p. administration of ketamine. The values represent the mean + SEM (*n* = 12–15 mice per group). ^^^*P* < 0.001 versus dexamethasone (Bonferroni’s post hoc test), ^###^ *P* < 0.001 versus dexamethasone and respective antidepressants (Bonferroni post hoc test), ***P < 0.001 versus control (*t*-test or Bonferroni’s post hoc test)

A single injection of an inhibitory dose of dexamethasone (64 μg/kg) was not able to abolish the antidepressant effect of imipramine (5 mg/kg), zinc (10 mg/kg), and imipramine-zinc combination (2.5 and 5 mg/kg, respectively) given once a day for 14 consecutive days (one-way ANOVA: *F*_2,42_ = 25.62, *P* < 0.0001 and *F*_2,42_ = 19.68, *P* < 0.0001 for imipramine and zinc, respectively; two-way ANOVA: *F*_1,56_ = 59.54, *P* < 0.0001 for imipramine-zinc interaction; Fig. 3b). The imipramine-zinc combination (2.5 and 5 mg/kg, respectively) was not active in the naïve mice (Fig. 3a).

### Influence of a chronic administration of imipramine and zinc given in combination with ketamine on a single injection of dexamethasone in the FST

An antidepressant effect of a single injection of ketamine (30 mg/kg) lasted shorter than 14 days (*t*-test: t_28_ = 1.045, *P* = 3.049). Neither imipramine (2.5 mg/kg/day) nor zinc (5 mg/kg/day) administered for 14 consecutive days at the sub-active doses selected o the naïve mice potentiated and/or prolonged the activity of ketamine, which was illustrated in Fig. 3b (two-way ANOVA: *F*_1,56_ = 2.62, *P* = 0.1112 and *F*_1,56_ = 1.43, *P* = 0.2373 for imipramine-ketamine and zinc-ketamine interactions, respectively).

### The influence of a single administration of dexamethasone, imipramine, zinc, ketamine, and their combinations on the spontaneous locomotor activity of mice

None of the tested agents given alone or in combinations according to the schemes applied in the Experiments 2, 3 and 4, influenced the locomotor activity of animals (*P* > 0.05, data not shown).

## Discussion

According to the literature (e.g., Brown et al. 1999), dexamethasone can induce a variety of psychiatric symptoms ranging from depression to mania and affective psychosis. Acute administration of this glucocorticosteroid causes neuronal death (accompanied by extensive sublethal neuronal damage) of granule cells in the dentate gyrus, pyramidal neurons in the CA1 and CA3 subfields of the hippocampus and striatopallidal neurons in the dorsomedial caudate putamen (Hassan et al. 1996; Haynes et al. 2001). Hippocampus and striatum are the brain areas that show morphological abnormalities in patients with mood disorders (Bremner et al. 2000; Rogers et al. 1998). Moreover, Haynes et al. (2004) reported the chronic (but not acute) pretreatment with different classes of antidepressants (but not with the psychoactive agents devoid of antidepressant potential) attenuated dexamethasone-induced neuronal damage. Then again, we previously demonstrated that both single and repeated administration of dexamethasone were able to modify the activity of antidepressant drugs from various pharmacological groups (Wróbel et al. 2014).

In the present study, the dexamethasone-injected mice subjected to the Porsolt test spent significantly more time being immobile compared to the vehicle-treated animals, which was indicative of their depressive behavior and appeared to be in line with the outcomes of our preceding experiments (Wróbel et al. 2014) and with the results obtained by Sigwalt et al. (2011). Just like in the repeated corticosterone injection paradigm, the observed effect was dose-dependent (Gregus et al. 2005; Johnson et al. 2006), but in our case, it appeared after both single and repeated administrations. Nevertheless, basing on the obtained results we are able to agree with the other authors (Gregus et al. 2005; Johnson et al. 2006) that the amount of the glucocorticoid and duration of treatment seems to be crucial determinants for the expression of a depressive phenotype in the forced-swim test. The results presented herein have not been confounded by the altered activity level of the tested animals, since the dexamethasone-treated mice showed no significant differences in overall spontaneous locomotor activity. For the same reason, it is highly unlikely that an increase in the immobility time observed in the FST could only be a simple consequence of weight loss in animals (Johnson et al. 2006; Marks et al. 2009; Zhao et al. 2008). Sigwalt et al. (2011) also emphasized that animals’ response to dexamethasone treatment seemed not to be influenced by their motor dysfunctions.

Though the further studies providing the biochemical explanation of the outcomes of our studies need to be performed, Li et al. (2014) have suggested that the behavioral abnormalities at juvenile and adult mice observed after neonatal dexamethasone exposition, may be the consequence of abnormalities in glutamatergic and GABAergic neurotransmissions. Kamphuis et al. (2003) found that exposure to dexamethasone during the neonatal period leads to a reduction in the GluN2B subunit levels of the NMDA receptor complex in hippocampus which may in the later life be implicated in the development of psychiatric diseases.

The outcomes of multiple pre-clinical and clinical studies have indicated the role of zinc in mood disorders. Apart from its own antidepressant-like properties demonstrated in animal tests and models (FST, TST) (Kroczka et al. 2000; Rosa et al. 2003), olfactory bulbectomy (Nowak et al. 2003), chronic unpredictable stress (Cieślik et al. 2007), and chronic mild stress (Sowa-Kućma et al. 2008), zinc also potentiated the activity of fluoxetine, paroxetine, citalopram, and bupropion (Cunha et al. 2008; Szewczyk et al. 2009). Zinc as an antagonist of the NMDA receptor complex and an agonist of the adenosine A1 and A2 receptors (Amico-Ruvio et al. 2011; Lobato et al. 2008) modulates glutamatergic, GABAergic, and glycinergic neurotransmissions (Mocchegiani et al. 2005) and may activate metabotropic GPR39 receptor (Holst et al. 2007). Special attention has been paid to connection between zinc deficiency and development of depression-like behavior in animals (Młyniec et al. 2012) and its correlation with hyperactivation of the HPA axis (Młyniec et al. 2012; Takeda et al. 2012; Watanabe et al. 2010). It has been shown that zinc-deficient diet induced a high serum corticosterone level in rats (Takeda et al. 2012), which may in turn be linked to glutamate accumulation and its excitotoxicity (Takeda and Tamano 2010). On the other side, corticosterone has been able to exert hippocampal zinc dyshomeostasis (Takeda and Tamano 2012). In our experiments, it appeared that the triple injections (i.e., 24, 5 and 1 h before the behavioral tests) of the lowest antidepressant dose of zinc (selected on the naïve mice) were potent enough to abolish the inhibitory effect induced by a 14-day treatment with dexamethasone at a daily dose of 16 μg/kg but an acute inhibitory dose of this glucocorticoid did not manage to reverse an antidepressant action of zinc given to animals for 2 weeks. Moreover, we noticed a stronger antidepressant effect of combination of zinc and imipramine in the repeatedly dexamethasone-injected animals but not in the naïve ones. The synergistic effect of these agents with the antidepressant potential was not recorded when given chronically to naïve mice in the sub-effective doses for 14 consecutive days. Nevertheless, such a combination exerted an effect strong enough to prevent the inhibitory activity of a single active dose of dexamethasone (i.e., 64 μg/kg). According to literature data, a repeated treatment with zinc alone or in combination with imipramine induces a significant increase in the BDNF mRNA level in the chronically stressed animals (Cieślik et al. 2011).

Ketamine, the other compound investigated in our experiment that acts through the glutamatergic neurotransmission, belongs to the noncompetitive NMDA receptor antagonists. Though its efficacy and safety profile still needs further confirmation, ketamine has appeared to be effective in patients with major depression, including the refractory cases (Murrough et al. 2013; Rao and Andrade 2010). The antidepressant activity of ketamine along with the mechanisms responsible for this effect have been studied in different animal models of depression, including the repeated corticosterone-injected rodents (Aan Het et al. 2012; Koike et al. 2013; Krystal et al. 2013). Koike et al. (2013) found that a subanesthetic dose of ketamine reduced the increased immobility time of the repeated corticosterone-injected rats. We demonstrated that a single administration of the lowest antidepressant dose of ketamine (selected on the naïve mice) suppressed inhibitory behavior induced by the chronic exposure to dexamethasone (16 μg/kg/day), while the acute or sub-chronic injections of ketamine-imipramine and ketamine-zinc combinations appeared to exert a synergistic effect in the repeatedly dexamethasone-injected animals but not in the naïve ones. Similar to our results, Li et al. (2014) reported that another NMDA receptor antagonist (i.e., Ro 63-1908, that binds specifically to the GluN2B subunit) prevented the depression-like behavior in juvenile mice after neonatal dexamethasone exposure. The authors presumed that the observed depressive behavior of juvenile animals could be a consequence of the alterations in glutamatergic transmission induced by the glucocorticoid.

According to the literature (Aan Het et al. 2012; Krystal et al. 2013), the clinical benefits of a single dose of ketamine may last as briefly as 1 or 2 days or as long as more than 2 weeks. In experiments performed by Zhang et al. (2014) a single dose of R-ketamine produced rapid and long-lasting (7 days) antidepressant effects in juvenile mice exposed neonatally to dexamethasone. The authors have suggested that antagonism of the NMDA receptor complex may promote a rapid antidepressant action of R-ketamine but most probably, it is not responsible for the longevity of the antidepressant effect. Li et al. (2014) provided further evidence of the rapid onset and enduring antidepressant effects of ketamine, which managed to ameliorate anhedonia in juvenile mice after neonatal dexamethasone exposure. The antidepressant effects were recorded at 46 h after a single ketamine dose and the ketamine-induced increase in sucrose intake persisted for 8 days. The outcomes of our experiments did not confirm a 2-week persistence of an antidepressant effect of a single dose of ketamine. It was not potentiated and/or prolonged by imipramine or zinc, either.

## Conclusion

Our findings indicate that the repeated dexamethasone injection procedure may constitute a potential model to study the expression of depressive symptomatology influenced by glucocorticoids. However, the confirmatory studies are required. First of all, the procedure should also be assessed in females, since it has been proven that males and females may respond in a different manner in behavioral models, and males may be more susceptible to the behavioral and neural consequences of repeated stress (Kalynchuk et al. 2004). Moreover, it would be interesting to compare the effects of the repeated dexamethasone injection observed in juvenile and adult subjects. At last, our results further support the theory of interplay between glutamatergic neurotransmission and the chronic or excessive exposition to glucocorticoids in behavioral alterations relevant to depressive symptoms.
